# Treatment of Hepatocellular Carcinoma with Immune Checkpoint Inhibitors and Applicability of First-Line Atezolizumab/Bevacizumab in a Real-Life Setting

**DOI:** 10.3390/jcm10153201

**Published:** 2021-07-21

**Authors:** Maria Corina Plaz Torres, Quirino Lai, Fabio Piscaglia, Eugenio Caturelli, Giuseppe Cabibbo, Elisabetta Biasini, Filippo Pelizzaro, Fabio Marra, Franco Trevisani, Edoardo G. Giannini

**Affiliations:** 1 Gastroenterology Unit, Department of Internal Medicine, IRCCS—Ospedale Policlinico San Martino, University of Genoa, 16132 Genoa, Italy; mariacorina.plaztorres@edu.unige.it; 2 Hepatobiliary and Organ Transplantation Unit, Umberto I Polyclinic of Rome, Sapienza University of Rome, 00185 Rome, Italy; quirino.lai@uniroma1.it; 3 Internal Medicine Unit, Department of Medical and Surgical Sciences, IRCCS Azienda Ospedaliero—Universitaria di Bologna, 40138 Bologna, Italy; fabio.piscaglia@unibo.it; 4 Gastroenterology Unit, Belcolle Hospital, 01100 Viterbo, Italy; e.caturelli@tiscali.it; 5 Gastroenterology and Hepatology Unit, Department of Health Promotion, Mother and Child Care, Internal Medicine and Medical Specialties (PROMISE), University of Palermo, 90133 Palermo, Italy; giuseppe.cabibbo@policlinico.pa.it; 6 Infectious Diseases and Hepatology Unit, Azienda Ospedaliero—Universitaria di Parma, 43126 Parma, Italy; ebiasini@ao.pr.it; 7 Gastroenterology Unit, Department of Surgery, Oncology and Gastroenterology, University of Padova, 35128 Padova, Italy; filippo.pelizzaro@unipd.it; 8 Internal Medicine and Hepatology Unit, Department of Experimental and Clinical Medicine, University of Firenze, 50134 Firenze, Italy; fabio.marra@unifi.it; 9 Medical Semeiotics Unit, Department of Medical and Surgical Sciences, IRCCS Azienda Ospedaliero—Universitaria di Bologna, 40138 Bologna, Italy; franco.trevisani@unibo.it

**Keywords:** liver cancer, systemic treatment, immunotherapy, real-world, unresectable hepatocellular carcinoma

## Abstract

Immune checkpoint inhibitors (ICIs) are the new frontier for the treatment of advanced hepatocellular carcinoma (HCC). Since the first trial with tremelimumab, a cytotoxic T-lymphocyte-associated protein 4 inhibitor, increasing evidence has confirmed that these drugs can significantly extend the survival of patients with advanced hepatocellular carcinoma (HCC). As a matter of fact, the overall survival and objective response rates reported in patients with advanced HCC treated with ICIs are the highest ever reported in the second-line setting and, most recently, the combination of the anti-programmed death ligand protein-1 atezolizumab with bevacizumab—an anti-vascular endothelial growth factor monoclonal antibody—demonstrated superiority to sorafenib in a Phase III randomized clinical trial. Therefore, this regimen has been approved in several countries as first-line treatment for advanced HCC and is soon expected to be widely used in clinical practice. However, despite the promising results of trials exploring ICIs alone or in combination with other agents, there are still some critical issues to deal with to optimize the prognosis of advanced HCC patients. For instance, the actual proportion of patients who are deemed eligible for ICIs in the real-life ranges from 10% to 20% in the first-line setting, and is even lower in the second-line scenario. Moreover, long-term data regarding the safety of ICIs in the population of patients with cirrhosis and impaired liver function are lacking. Lastly, no biomarkers have been identified to predict response, and thus to help clinicians to individually tailor treatment. This review aimed to summarize the state of the art immunotherapy in HCC and, by analyzing a large, multicenter cohort of Italian patients with HCC, to assess the potential applicability of the combination of atezolizumab/bevacizumab in the real-life setting.

## 1. Introduction

Hepatocellular carcinoma (HCC) is one of the leading causes of cancer-related mortality worldwide, with approximately 800,000 deaths per year and an estimated increase to more than 1 million deaths by 2030 [1]. HCC arises predominantly in the context of liver cirrhosis, but also can be diagnosed in a not negligible proportion of patients without cirrhosis suffering from non-alcoholic steato-hepatitis who carry additional metabolic and genetic risk factors [2,3,4,5]. In the past decades, the *armamentarium* for the systemic treatment of advanced HCC was limited to the anti-vascular endothelial growth factor (VEGFR), multi-target-tyrosine kinase inhibitor (TKI) sorafenib. This drug determined a significant—though modest—survival benefit in two Phase III trials and remained the sole first-line treatment option for about 10 years, during which neither an alternative drug nor effective second-line therapies became available for patients who progressed during—or were intolerant to—sorafenib [6,7]. As a fact, lenvatinib (a TKI targeting VEGFR) became an effective alternative to sorafenib as first-line therapy for HCC in 2018, while regorafenib, cabozantinib, and ramucirumab only recently have been approved in the second-line setting [8]. With the advent of second-line treatments, the survival of patients with advanced HCC has significantly improved, with a proportion (approximately 20%) of patients reaching survival times of about 2 years with the sequential use of sorafenib-regorafenib [9]. These patients, however, belong to a small subgroup of patients who, maintaining an optimal liver function, are eligible for sequential treatment and tolerate the adverse effects of the anti-neoplastic agents [9].

In this scenario, immunotherapy has emerged as an additional promising approach potentially able to obtain even longer survival times. Research in this field is steadily increasing, also fueled by the positive results obtained in other cancer types and by the evidence of efficacy demonstrated in both first- and second-line settings [10,11,12]. The most recent Phase I/II trials have shown a clinically meaningful survival increase in the second-line setting for the programmed cell death protein 1 (PD-1) inhibitors nivolumab and pembrolizumab [12]. Hence, these agents have been granted accelerated conditional approval for sorafenib-experienced patients in the US, while the European Medicines Agency (EMA) maintains a more cautious attitude in approving these ICIs for the treatment of HCC. Indeed, subsequent Phase III trials testing nivolumab versus sorafenib as first-line treatment, and pembrolizumab versus placebo in second-line treatment, failed to meet their primary survival endpoints [13,14]. This notwithstanding, the results from trials testing the combination of immune checkpoint inhibitors (ICIs) with other agents, among which VEGFR-targeted therapies obtained very encouraging results, so that the combinations of pembrolizumab plus lenvatinib as well as atezolizumab (monoclonal antibody against PD-L1) plus bevacizumab (monoclonal antibody against VEGF) have both received breakthrough therapy designation from the US Food and Drug Administration (FDA). Actually, in a recent Phase III trial, the latter overperformed compared to sorafenib as first-line treatment of advanced HCC in terms of both overall (OS) and progression-free survival (PFS) [15]. Therefore, atezolizumab plus bevacizumab has been approved as the first-line treatment option for advanced HCC, thus becoming the standard of care for these patients. 

Overall, the results from the trials testing ICIs alone or in combination, or combined with other agents, suggest that ICIs alone are not the best option for the treatment of HCC, while combined treatments are safe and highly effective. As such, immunotherapy-based treatments will probably soon change the landscape of advanced HCC therapy. In this review, we summarize the state of the art immunotherapy in advanced HCC, with a particular focus on the combination of atezolizumab plus bevacizumab, by assessing in a large cohort of Italian patients with HCC the potential applicability of this regimen to the real-life setting.

## 2. Approved Treatments for HCC before the “Era” of Immune Checkpoint Inhibitors

Until the approval of sorafenib in 2008, no systemic treatment was available for advanced HCC [6]. Sorafenib, an orally active multi-target TKI targeting different cell surface tyrosine kinases (e.g., VEGFR-1, -2, and -3 and platelet-derived growth factor (PDGFR)-β), at the dose of 400 mg twice daily, significantly improved OS in patients with HCC not amenable to surgery and locoregional procedures, who had well-preserved liver function (97% Child–Pugh A) and Eastern Cooperative Oncology Group (ECOG) performance status (PS) ≤ 2 [6]. The median OS was 10.7 months in the sorafenib group and 7.9 months in the placebo group (*p* < 0.001), whereas the median time to radiologic progression (TTP) was 5.5 months in the sorafenib arm versus 2.8 months in the placebo arm (*p* < 0.001). Of note, the median OS of patients with the Barcelona Clinic Liver Cancer (BCLC) staging system stage B HCC treated with sorafenib was 15–20 months, a finding confirmed by subsequent post-marketing studies [16,17]. In the following years, several drugs were tested against sorafenib in the first-line setting, failing to demonstrate superiority to this drug, so that sorafenib remained the sole effective systemic treatment available for HCC until 2018, when lenvatinib, an oral TKI with a biologic action similar to sorafenib, showed non-inferior OS as compared to sorafenib in the REFLECT trial, and was therefore approved as an alternative to this drug in the first-line setting [8]. Again, patients included in this trial belonged to a selected group of subjects with well-preserved liver function (Child–Pugh class A) and ECOG PS ≤ 1, while those with extensive tumor burden (≥50% of the liver), bile duct invasion, or invasion of the main portal vein were excluded. Forest plots for OS revealed that lenvatinib was more effective than sorafenib in patients with baseline AFP ≥ 200 ng/mL (Hazard ratio (HR), 0.78; 95% confidence interval (95%CI), 0.63–0.98) and less effective in patients without macrovascular invasion/extrahepatic spread and those enrolled in the Western area. Secondary endpoints (PFS, TTP, objective response rate (ORR)) were significantly and remarkably better with lenvatinib, suggesting that these surrogate endpoints poorly predict OS in HCC patients treated with these drugs.

As far as the second-line setting is concerned, regorafenib, an oral TKI targeting VEGFR-2, VEGFR-3, TIE-2, PDGFR, fibroblast growth factor receptor (FGFR)-1, and the mutant oncogenic kinases KIT, RET, and B-RAF, was the first agent able to provide a significant survival benefit in patients with tumor progression on sorafenib [18]. Compared with placebo, regorafenib improved OS with a HR of 0.63 (95%CI, 0.50–0.79; *p* < 0.0001). It has to be emphasized that this study enrolled patients who progressed on sorafenib but tolerated the drug (≥400 mg/day for ≥20 of last 28 days of treatment) and had Child–Pugh class A liver function. Median survival was 10.6 months (95%CI, 9.1–12.1) for the regorafenib group versus 7.8 months (95%CI, 6.3–8.8) for the placebo group [18]. Interestingly, the treatment sequence of the sorafenib-regorafenib group was able to determine an OS of 26 months from the start of sorafenib treatment versus 19.2 months in the sorafenib-placebo group [18]. This survival time is comparable with that of patients with intermediate stage HCC undergoing trans-arterial chemo-embolization (TACE), suggesting that in a well-selected subgroup of patients the sequential treatment with TKIs may significantly improve prognosis as compared to the standard of care [9].

Other drugs that have shown efficacy in placebo-controlled trials and have consequently been approved as second-line treatment options for HCC are cabozantinib and ramucirumab [19]. Cabozantinib is an oral TKI targeting MET in addition to VEGFR2. The CELESTIAL trial was a global Phase III trial testing cabozantinib in patients with HCC progression on sorafenib [20]. It also included patients who had received up to two prior therapies for advanced-stage HCC. The study was stopped after a second interim analysis, which revealed a median OS of 10.2 months in the cabozantinib versus 8.0 months in the placebo group (HR, 0.76; 95% CI 0.63–0.92; *p* = 0.0049). Approximately 72% of patients had received only prior sorafenib treatment and, in this subpopulation, median OS was even longer, being 11.3 months in patients in the cabozantinib group versus 7.2 months in the placebo group (HR, 0.70; 95% CI, 0.55–0.88) [20].

Ramucirumab is an anti-VEGFR2 monoclonal antibody, and its utility in subjects with advanced HCC emerged from the double-blind, Phase III REACH-2 trial comparing ramucirumab versus placebo as second-line treatment in patients progressing on sorafenib and with baseline AFP ≥ 400 ng/mL [19]. This study was designed on the basis of the results of the REACH trial that failed to demonstrate an OS advantage with ramucirumab as compared to the placebo, but in a post-hoc analysis showed a benefit of the drug—albeit small—in prolonging OS (8.5 months with ramucirumab versus 7.3 months with placebo (HR, 0.71; 95% CI, 0.53–0.95; *p* = 0.0199)) among patients with baseline AFP ≥ 400 ng/mL [21]. Ramucirumab is therefore the first agent with a biomarker-driven use for patients with HCC progression on sorafenib [22].

In summary, sorafenib and lenvatinib are the TKIs that have long been in use for the front-line treatment of advanced HCC, providing a median extension of survival of about 3 months compared to the placebo. The survival benefit for patients eligible for second-line treatment with regorafenib/cabozantinib or ramucirumab, although significant, still remains modest. Hence, novel treatments targeting different tumorigenic pathways have been studied and others are still under investigation with the aim of further improving the outcomes of these patients. In this context, ICIs have gained excellent results.

## 3. The Advent of Immune Checkpoint Inhibitors

Despite the benefit in OS with sequential TKI treatment, the prognosis of patients with advanced HCC remains poor [9,23]. The reasons for this include, besides the sub-optimal tumoricidal activity of these drugs, the progression of the underlying liver disease, the advanced median age of this cohort of patients (approximately 70 years), and the presence of substantial comorbidities, which are very frequent in these subjects and, overall, make them a particularly vulnerable cohort [24].

In this context, ICIs have increasingly been investigated in the last years, with extremely encouraging results both in the first- and second-line setting, further boosting a rising number of clinical trials using ICIs alone or combined with other anti-tumoral drugs or with locoregional treatment. The rationale for the use of ICIs in HCC relies on the fact that HCC arises in a context of chronic inflammation and an altered tumor microenvironment, with the presence of tumor-infiltrating lymphocytes expressing PD1, which is a recognized key enabling factor beyond tumor cell-intrinsic molecular aberrations [25,26,27]. Moreover, the presence of PD1-expressing lymphocytes in HCC samples has been correlated with this outcome [28,29]. In this regard, Sia et al. have recently proposed a novel HCC classification based upon the tumor immune status: according to this classification, about 30% of HCCs could be categorized into an ‘immune class’, with high levels of immune cell infiltration, expression of PD-1 and/or PD-L1, activation of interferon-γ signaling, and markers of cytolytic activity [30]. Within this class, two distinct subclasses have been identified: the ‘active immune’ and the ‘exhausted immune’ classes, characterized by markers of an adaptive T-cell response or of an exhausted immune response, respectively [30]. The latter subclass is the ideal target of immunotherapy. The in-depth description of the molecular mechanisms involved in the tumor microenvironment of HCC is beyond the aim of this article, but it is worth pointing out that interactions between cancer cell antigens and the antigen-presenting cells lead to a priming of T-cells and their eventual migration into the tumor microenvironment. Physiologically, the T-lymphocytes’ recognition of neoplastic antigens is followed by a T-cell-mediated killing of cancer cells [31]. This process is finely modulated at a local and general level by several mechanisms including immune checkpoints, which play a pivotal role in such modulation, as they suppress T-cell activity to inhibit eventual over-activation of the immune system and maintain self-tolerance. Thus, immune checkpoints physiologically prevent hyperimmune responses leading to tissue damage. Malignancies exploit these molecular mechanisms (immune checkpoints) to escape from the immune system recognition. In other words, ICIs act as anti-neoplastic agents by inhibiting negative feedback pathways of the immune system that mediate immune escape.

The most largely studied immune checkpoints are PD-1 and cytotoxic T-lymphocyte-associated protein 4 (CTLA-4). The pathological activation of PD-1 by its ligands, in particular PD-L1, expressed by cancer cells, can result in the immune escape of the tumor [32,33]. CTLA-4, which is mainly expressed on T-cells, regulates T-cell activity in physiological conditions, preventing an excess in T-cell responses and a hyperactivation of the immune response. Inversely, in pathological (neoplastic) conditions, CTLA-4 activation inhibits in the activation, proliferation, and production of tumor antigen-activated T-cells in the tumor microenvironment [32,33]. In the HCC tumor microenvironment, T-regulators (T-regs) express both CTLA-4 and PD-1 [28,32].

## 4. Immune Checkpoint Inhibitors in HCC

**Figure 1:**
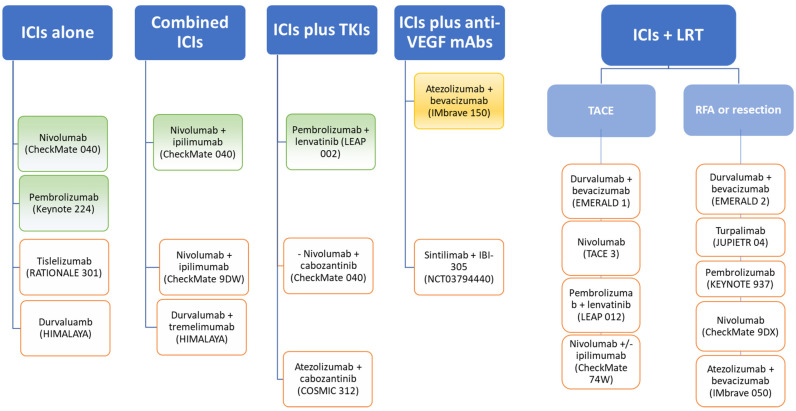
Possible HCC treatments with ICIs. Atezolizumab plus bevacizumab has been approved as a first-line treatment, whereas nivolumab with or without ipilimumab and pembrolizumab gained FDA approval as second-line treatments. Selected Phase III trials (orange squares) are testing ICIs alone or in combination or combined with other agents in the first and second-line setting, and in the adjuvant and neo-adjuvant setting as well.

Tremelimumab, a CTLA-4-blocking monoclonal antibody, was the first ICI showing benefits in the treatment of HCC. This agent was tested in 2013 by Sangro et al. in a Phase II open-label trial that enrolled 21 patients with advanced HCC who were either sorafenib-naïve (76.2%) or -experienced, and a significant proportion of them were classified as Child–Pugh class B (43%) [10]. The positive results in terms of both safety and anti-tumor activity (partial response rate (PRR) 17.6%; disease control rate (DCR) 76.4%; TTP 6.48 months (95%CI, 3.95–9.14)), were instrumental in stimulating the research in immune checkpoint blockade in both first- and second-line treatment of HCC. In the last years, the effects of ICIs in HCC have been tested alone or in combination with other ICIs or combined with agents targeting the VEGFR. Currently available immunotherapy-based regimens and those under Phase III clinical investigation are summarized in Figure 1.

### 4.1. Immune Checkpoint Inhibitors in Monotherapy

Following the encouraging results of the Phase II tremelimumab study, nivolumab, a monoclonal antibody targeting PD1, demonstrated a single-agent activity in the Phase Ib/II open-label, non-comparative, Checkmate 040 trial [11]. The initial trial included 262 sorafenib-naïve and -experienced patients assigned to a dose-escalation (48 subjects) or to a dose-expansion (214 subjects) phase. In the dose-expansion phase, the investigator-assessed overall ORR was 20%, with 3 complete responses (CR) and 39 partial responses (PR). Particularly, ORR was 22.5% for sorafenib-naive and 18.7% for sorafenib-experienced patients. Median OS was 29 months for sorafenib-naïve group and 15 months for the sorafenib-experienced group. The most impressive was the duration of response of 9.9 months amongst patients who had an objective response, which led the US FDA to grant accelerated approval to nivolumab as second-line therapy for patients with advanced-stage HCC previously treated with sorafenib [11]. In this subgroup, the ORR confirmed by blinded independent central review was 14.3% by Response Evaluation Criteria In Solid Tumors (RECIST) 1.1 and 18.2% by modified RECIST (mRECIST) criteria. Of note, the median duration of response was the longest ever seen in a second-line setting: 16.6 months [34,35]. However, the expectations raised by the results of this study were disappointed in a subsequent Phase III randomized trial (CheckMate-459) testing nivolumab versus sorafenib, as the anti-PD1 agent failed to demonstrate superiority as compared to the TKI [14]. Still, the study results confirmed clinically meaningful improvements in OS (16.4 versus 14.7 months), ORR (15% for nivolumab versus 7% for sorafenib), and CR (14 versus 5 patients). Moreover, nivolumab demonstrated a favorable safety profile, consistent with previous reports and, of particular interest, the quality of life was better in the nivolumab treatment arm [14]. The long survival of the sorafenib arm (median OS of about 15 months) was an unexpected outcome that negatively impacted the study results and that probably reflects the improved tailored management of patients with advanced HCC in the last decades, as well as physicians’ familiarity with the TKI.

Another ICI that has been tested with favorable outcomes in monotherapy for advanced HCC is pembrolizumab, a monoclonal antibody targeting PD-1. Promising results came from the Phase II trial KEYNOTE 224, which showed good responses (ORR 17%, DCR 61%) and a good safety profile of pembrolizumab in patients who were intolerant to, or progressed under, sorafenib [12]. These results prompted Finn et al. to conduct the KEYNOTE-240 trial enrolling 413 patients who failed sorafenib and who were randomized 2:1 to pembrolizumab or placebo [13]. The survival in the pembrolizumab arm was among the highest ever reached in the second-line setting, being approximately 14 months (95%CI, 11.6–16.0) for pembrolizumab versus 10 months (95%CI, 8.3–13.5) for placebo (HR, 0.781; 95%CI, 0.611–0.998; *p* = 0.0238). Nevertheless, even this study failed to reach statistical significance due to the long survival of the control arm, reflecting once more the advances in the clinical management of advanced HCC. The safety profile of the drug was good, confirming the positive results of the Phase II study and the previous experience with nivolumab.

Despite the apparently “negative” results of these studies, likely due to issues related to their design requesting an overwhelming superiority of the tested ICIs over sorafenib, several positive aspects capturing the attention of researchers and clinicians were the overall objective response to nivolumab and pembrolizumab in 15–20% of cases, the durable antitumor responses, and the long-term OS in responding patients. Based on these peculiar results, the FDA granted conditional approval for these ICIs in the second-line setting.

Currently, results from the ongoing Phase III non-inferiority trial testing tislelizumab, a monoclonal antibody targeting PD-1, versus sorafenib (RATIONALE-301 trial) and those of the Phase III HIMALAYA study, testing durvalumab—an anti-PD-L1 monoclonal antibody—alone or in combination with tremelimumab versus sorafenib, are eagerly awaited [36].

As far as the safety profile of ICIs is concerned, the results of the pilot study by Sangro et al. on tremelimumab and those of the CheckMate and Keynote trials showed reassuring safety profiles for these agents, coherent with previous reports testing the use of these drugs in other cancer types [10,11,12,37]. As compared with the standard of care (i.e., sorafenib and lenvatinib), ICIs are generally better tolerated and have comparable or even lower rates of toxicity. The pathophysiology of adverse events (AEs) occurring during immunotherapy is related to their mechanism of action as the inhibition of physiological immune checkpoints may trigger immune-related AEs (irAEs) targeting the skin, gut, thyroid, adrenal glands, lung, and the liver itself, which may be a particularly worrisome complication in a population with an already impaired liver function [38,39]. Most frequent any grade AEs in patients treated with ICIs for other cancer types are skin AEs (rash and pruritus), colitis, hyper- or hypothyroidism, hepatitis, and pneumonitis. Skin AEs occur in about 13–35% of cases, being grade >3 only in a minority of cases (<3%) [38,40]. Grade 1 and 2 skin AEs are usually easily managed with emollients, oral anti-histamines, and topical steroids, whereas grade ≥ 3 reactions require oral corticosteroids administration and the discontinuation of the immunotherapy until the skin AE has reverted to grade 1 [38]. Thyroid dysfunction has been reported in a variable proportion of cases (5–20%), but these events are rarely severe and rarely require treatment discontinuation or hormonal replacement treatment or corticosteroids administration [38]. The frequency of colitis ranges from 2% to 22% [38,40], being more frequent and severe in patients treated with anti-CTLA4 agents [38,40]. Again, the incidence of high-grade colitis is very low, being around 1–2% [40]. Patients with non-severe diarrhea should be treated with anti-diarrheal, fluid replacement, and electrolytes; conversely, patients with grade ≥ 3 diarrhea or persistent grade 2 diarrhea should discontinue ICIs and receive intravenous (i.v.) corticosteroids. In case of lack of response to corticosteroids, infliximab should be prescribed [38]. Pneumonitis occurs in 2–4% of patients, with grade ≥ 3 events representing only 1% to 2% of cases [38,40], and the frequency of fatal pneumonitis and that of treatment discontinuation (due to this AE) are extremely low (0.2% and 0.2–4%, respectively) [38]. In the case of documented or high suspicion of immune-related pneumonitis, immunosuppressive treatment should be started immediately. In grade 1 to 2 pneumonitis, treatment consists of oral steroids (prednisone 1 mg/kg daily), whilst patients with grade 3 to 4 pneumonitis should be hospitalized and treatment should consist of high-dose i.v. corticosteroids. In these severe cases, immunotherapy should be permanently discontinued. With regards to the occurrence of treatment-related hepatitis, which occurs in a proportion of 5% to 10% of patients (among which 1–2% are grade 3) [38,40], in the presence of grade ≤ 2 transaminases elevation, checkpoint inhibitor therapy should be withheld and transaminases and bilirubin should be measured twice weekly. Persistent grade 2 elevation lasting longer than 2 weeks, after having ruled out other causes, should be treated with corticosteroids at a dose of 1 mg/kg/day (methyl)prednisolone or equivalent. Upon improvement, re-challenge with ICIs may be attempted after corticosteroid tapering. In the absence of improvement despite the initiation of corticosteroids, the dose should be increased to 2 mg/kg/day of (methyl)prednisolone or equivalent and checkpoint inhibitor therapy should permanently be discontinued [38]. In the instance of grade 3 or 4 transaminase or total bilirubin elevation, checkpoint inhibitor therapy should be permanently discontinued, and corticosteroids started at 1–2 mg/kg/day (methyl)prednisolone or equivalent. If the absence of response to corticosteroids within 2–3 days, mycophenolate mofetil should be added at 1000 mg twice daily. If no improvement is seen, liver biopsy should be considered. However, ICI-related hepatitis usually resolves within 4–6 weeks with appropriate treatment; therefore, if no improvement is detected in this time frame, other contributory causes should be reconsidered and the initial diagnostic work-up should be repeated.

Overall, the available evidence suggests that, although common, irAEs can be easily managed in most cases by delaying the subsequent scheduled administrations, and with the administration of corticosteroids in severe cases [39]. In HCC studies, approximately 90–98% of patients experienced any AE during treatment, with up to 50% of them being grade 3 or higher [10,11,12,13,14]. However, similar rates of AEs have been recorded in randomized controlled trials in the respective placebo arms as well [13]. With regards to treatment-related AEs, grade ≥ 3 AEs have been reported in approximately 20% of cases for nivolumab and pembrolizumab monotherapy [13,14]; among them, the most frequent AE in the Keynote-240 and CheckMate-040 studies was aminotransferase increase (about 4–5% and 6–10%, respectively) [11,13]. This event is of particular concern in patients with cirrhosis due to the potential deterioration of liver function and to the peculiar risk of corticosteroid-related AEs in these subjects. However, current data show that ICIs are safe in well-selected cohorts of patients with cirrhosis and preserved liver function (Child–Pugh class A), with no safety alerts as compared with patients without cirrhosis treated with ICIs for other cancer types [9,41]. The available evidence thus suggests that cirrhotic patients with HCC should not be at increased risk of liver irAEs, but close monitoring of liver function tests should be performed in cirrhotic patients treated with ICIs. Treatment-related serious AEs such as pneumonitis and colitis occurred in a minority of patients (<1%), as reported in the literature for immunotherapy in other cancer types [11,12]. Definite data on the safety and tolerability of ICIs in Child–Pugh class B patients, which represent a significant proportion of advanced HCC patients, are lacking. However, those from the CheckMate-040 trial are reassuring, since only 4 out of 49 patients with Child–Pugh class B reported treatment-related hepatic events, and only 2 of them needed treatment discontinuation [38]. Moreover, similar results regarding the safety of nivolumab and pembrolizumab in patients with Child–Pugh class B have been observed by Scheiner et al. in a real-life cohort of HCC patients [41]. Taken together, the available evidence suggests the safety profile of ICIs in the HCC population is good in selected cases with well-preserved liver function and that ICIs may be safely administered in Child–Pugh class B patients as well.

### 4.2. Dual Immune Chechpoint Blockade

Based on the hypothesis that anti-PD-1 and anti-CTLA4 agents may have a synergistic effect by inhibiting two different steps of the immune checkpoint system, combinations of anti-PD1 and anti-CTLA4 are underway. A Phase III trial with dual treatment with nivolumab plus ipilimumab, a CTLA-4 monoclonal antibody, in the first-line setting (CheckMate 9DW, NCT04039607) is underway. This trial was supported by the positive results observed in the cohort 4 (nivolumab plus ipilimumab) of the Checkmate-040 trial in the second-line setting [42]. In this study, patients were randomized 1:1:1 to either nivolumab 1 mg/kg plus ipilimumab 3 mg/kg, administered every 3 weeks (4 doses), followed by nivolumab 240 mg every 2 weeks (arm A); nivolumab 3 mg/kg plus ipilimumab 1 mg/kg, administered every 3 weeks (4 doses), followed by nivolumab 240 mg every 2 weeks (arm B); or nivolumab 3 mg/kg every 2 weeks plus ipilimumab 1 mg/kg every 6 weeks (arm C). Treatment combination had manageable safety, promising ORR, and durable responses. The arm A regimen showed the greatest benefits in terms of ORR (32% versus 27% and 29% in arms B and C, respectively) and OS (22.8 months (95%CI, 9.4—not reached) in arm A versus 12.5 months (95%CI, 7.6–16.4) in arm B and 12.7 months in arm C (95%CI, 7.4–33.0) [43]. Any grade treatment-related AE occurred in 94% of cases in arm A, 71% in arm B, and 79% of cases in arm C. Among them, 53% of patients in arm A, 29% of patients in arm B, and 31% of patients in arm C had grade 3 or 4 treatment-related AEs. Arm A also had higher rates of irAEs and irAEs leading to treatment discontinuation (18%), as compared with arms B and C (6% and 4%, respectively). Consequently, in arm A, 16% of patients stopped treatment: 6% of them due to treatment-related hepatitis, 6% due to pneumonitis, and 4% due to diarrhea/colitis [43]. However, most cases of patients presenting AEs continued treatment and the AEs resolved with standard management, while only 1 treatment-related death due to pneumonitis was reported (0.6%) [43]. Importantly, among patients who were re-challenged with nivolumab or ipilimumab after experiencing an irAE in any category, no patients experienced an event recurrence after the re-challenge [43]. Considering the outstanding OS and ORR obtained in arm A, these results suggest that nivolumab plus ipilimumab may provide improved efficacy in terms of ORR, and, potentially, of survival with an acceptable safety profile. Based on this evidence, this dual treatment received accelerated approval in the US as second-line treatment for HCC.

In the first-line setting, a Phase III trial (HIMALAYA) is testing the PD-L1 inhibitor durvalumab alone and in combination with tremelimumab, compared with sorafenib. This study was designed on the basis of the findings from a Phase I/II, randomized, open-label study that included patients progressing under, intolerant to, or refusing sorafenib [44]. Patients were randomized 1:2 to different tremelimumab plus durvalumab combinations, and safety was the primary endpoint. Patients assigned to the high-dose tremelimumab arm (i.e., tremelimumab 300 mg plus durvalumab 1500 mg 1 dose followed by durvalumab every 4 weeks) had the highest confirmed ORR (duration of response not reached) and longest OS (18.7 months (10.8—not reached)) [44]. Grade 3 or 4 treatment-related AEs rates were comparable to those occurring in the nivolumab plus ipilimumab trials, being 35% in the high-dose (300 mg) tremelimumab arm and 25% in the low-dose (75 mg) tremelimumab arm. Discontinuation of the study drug due to AEs was 10.8% and 6% in the high- and low-dose arm, respectively, but no deaths were attributed to treatment.

In summary, dual checkpoint blockade may improve OS in HCC patients, but consistent evidence is still scarce. As might have been expected, the trials testing ICIs in dual treatment reported higher rates of AEs in comparison with ICIs used in monotherapy, but in most cases, the safety profile was consistent in presentation and management with that of monotherapy. Taking into consideration the poor prognosis of patients with advanced HCC, the benefit/risk ratio may still favor the dual treatment strategy. Current trials with dual checkpoint blockade are reported in Table 1.

### 4.3. Immune Checkpoint Inhibitors Combined with Tyrosine Kinase Inhibitors

In addition to its well-known stimulating effect on angiogenesis, VEGF can promote immune evasion by directly and indirectly inhibiting infiltration and function of cytotoxic T-lymphocytes and increasing PD-1 expression on intra-tumoral CD8+ T-cells. In other words, the VEGF pathway is involved in the recruitment of immunosuppressive T-reg cells into the tumor. Thus, VEGF inhibition through TKIs or VEGFR-directed monoclonal antibodies might increase local antitumor immunity and favorably modify the immunosuppressive tumor microenvironment, thus enhancing the effects of ICIs [45]. On this basis, several Phase I/II trials testing combinations of anti-PD1/PD-L1 with anti-VEGFRs were undertaken and have already shown promising results in this research field, paving the way for Phase III trials that are currently in progress (Table 1) [46].

Among these studies, one trial tested the combination of nivolumab plus cabozantinib, with or without ipilimumab, reporting preliminary clinically meaningful responses [47]. As of today, the results of this study, which included 71 patients randomized to either nivolumab plus cabozantinib (*n* = 36) or nivolumab plus ipilimumab and cabozantinib (*n* = 35), are only partially available, and show that investigator-assessed ORR was comparable with that of nivolumab alone for the dual treatment arm (17%, 6 patients with PR) but reached 26% (9 patients with PR) in the triple treatment arm. The diseased control rate was good and similar in the two groups, being 81% for the dual treatment arm and 83% for the triple treatment arm. It is noteworthy that the median OS was not reached in either arm [47]. With regards to safety, grade 3 or 4 treatment-related AEs were observed in 42% of cases in the dual treatment arm and in 71% of cases in the triple treatment arm, leading to treatment discontinuation in 3% and 20% of patients, respectively. However, no new safety signals were observed in either arm. Based on these promising findings, complete and updated results of this trial are eagerly awaited.

Another combination that is currently under investigation in patients with advanced HCC is that of pembrolizumab plus lenvatinib, which, in a Phase Ib study, showed good results with a median OS of 22 months and a 46% confirmed ORR [48]. Hence, this combination has been granted a breakthrough therapy designation by the FDA for advanced HCC patients who are not amenable to locoregional treatment, and it is currently being tested in a Phase III, international, multicenter clinical study (LEAP-002).

### 4.4. Immune Checkpoint Inhibitors Combined with Anti-VEGFR Agents

Recently, Finn et al. tested the combination of atezolizumab, a monoclonal antibody targeting PD-L1, plus bevacizumab, an anti-VEGF monoclonal antibody, as a front-line treatment of advanced HCC. The trial (IMbrave-150) showed a clear superiority of the dual therapy over sorafenib [15]. The intention-to-treat population included 336 patients in the atezolizumab plus bevacizumab group and 165 patients in the sorafenib group. At the time of the primary interim analysis, the HR for death with atezolizumab plus bevacizumab as compared with sorafenib was 0.58 (95%CI, 0.42–0.79; *p* < 0.001). The reported 12-month OS was 67.2% (95%CI, 61.3–73.1) with atezolizumab plus bevacizumab versus 54.6% (95%CI, 45.2–64.0) with sorafenib. Median PFS was 6.8 months (95%CI, 5.7 to 8.3) and 4.3 months (95%CI, 4.0–5.6) in the respective groups (HR for disease progression or death: 0.59; 95%CI, 0.47–0.76; *p* < 0.001) [15]. Hypertension, proteinuria, and fatigue were the top three treatment-related AEs in the combination arm. Upper gastrointestinal bleeding, a known AE of bevacizumab and a main concern in patients with cirrhosis, occurred in 7% of patients in this group, which is well within the range of previous studies evaluating the use of bevacizumab in HCC [49,50]. Esophageal varices hemorrhage occurred in 2.4% of cases, but only 1.8% were grade ≥3 and less than 1% of cases needed treatment discontinuation. Of note, in this study, causality was reported only in <1% of patients [15]. In this respect, it is important to emphasize that patients intended to receive the combination of atezolizumab plus bevacizumab had undergone endoscopic variceal screening, as per the study protocol. Given the increased bleeding risk associated with bevacizumab, patients with gastro-esophageal varices at risk of bleeding received adequate prophylactic treatment, as must be done in standard care of cirrhotic patients with esophageal varices [51,52]. Increases in aminotransferases and pruritus were other common AEs attributable to atezolizumab but, again, only a few patients (0.6% of cases) needed to stop treatment and developed immune-mediated liver damage. The proportion of patients who discontinued any treatment component because of AEs was 15.5% in the atezolizumab plus bevacizumab group (7% discontinued both components) and 10.3% in the sorafenib group [15]. Overall, AEs leading to dose modification or interruption occurred in 49.5% of patients who received atezolizumab plus bevacizumab and in 60.9% of those who received sorafenib. Therefore, this study provided the first and strong—evidence of the benefit provided by combining an ICI and a VEGFR inhibitor for patients with advanced HCC, and its superiority over sorafenib has undoubtedly already changed the standard of care for these patients, where it has substituted sorafenib as first-line treatment in most cases. Nevertheless, as only patients with Child–Pugh class A were included in this study, which is standard practice in HCC trials, so no consistent data are available regarding efficacy and safety of this combination in patients with a greater impairment in liver function. To date, only one study has reported the outcomes for four Child–Pugh class B patients treated with atezolizumab plus bevacizumab in a Japanese cohort of patients [53]. Among these patients, all patients could be treated without the development of severe AEs until tumor progression and efficacy was comparable to that of Child–Pugh class A patients. These results are undoubtedly important, but further research in larger cohorts of patients is needed before a recommendation can be made for the use of this immunotherapy in patients with Child–Pugh class B liver function. However, we could argue that well selected patients with Child–Pugh class B7 liver function may be treated safely with atezolizumab plus bevacizumab but close monitoring of biochemistry and close clinical monitoring should be performed and patients should be informed that the benefit of this treatment in the Child–Pugh class B population still has to be determined.

The role of sorafenib and that of lenvatinib and, more in general, the treatment algorithms for the systemic treatment of HCC, will soon need to be reviewed in order to be optimized. Whether TKIs are going to be part of the second-line treatment alternatives, alone or in combination with other agents, is still unknown and extensive research is ongoing to try to adequately frame treatment sequences.

### 4.5. Immune Checkpoint Inhibitors Combined with Locoregional Treatments for HCC

**Figure 2:**
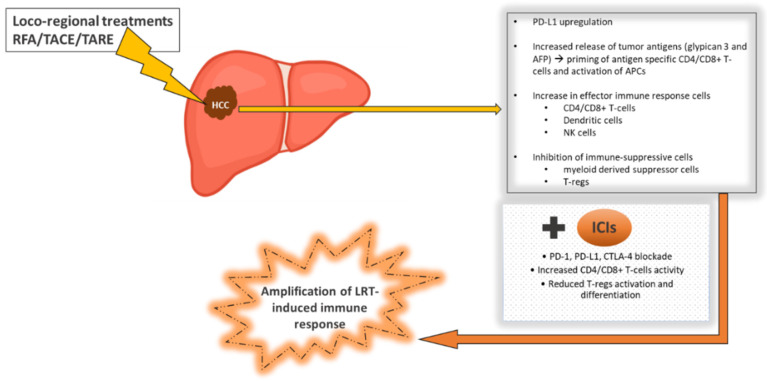
Locoregional treatments applied to hepatocellular carcinoma (HCC) induce immunological effects in the tumor microenvironment, which can be amplified by immune checkpoint inhibitors. After radiofrequency ablation (RFA) or trans-arterial chemo-embolization (TACE) or radio-embolization (TARE), necrosis of tumor cells induces increased tumor-antigen release, thus facilitating the recruitment and activation of cytotoxic T-cells and dendritic cells. These effects can be exploited by administering immune checkpoint inhibitors (ICIs) to transform an immunosuppressive microenvironment in an immune-supportive one, in which systemic therapies might be more effective.

To date, no systemic treatment tested in combination with locoregional treatments for HCC has demonstrated benefit in terms of OS. Conversely, ICIs might revolutionize the therapeutic panorama of early and intermediate stage HCC, thus achieving a role not only in the setting of palliative treatment, but also in the curative one. The rationale for their use in combination with radiofrequency ablation (RFA) and TACE relies on the fact that ablative and intra-arterial techniques indirectly induce a peripheral immune response that can enhance the effect of ICIs [54,55] (Figure 2). Namely, the RFA- and TACE-induced necrosis of tumor cells favors the release of tumor antigens and the activation of immune-mediated death of tumor cells, which, in turn, stimulate a peripheral systemic immune response that can potentially be amplified by immune checkpoint blockade [56,57,58,59,60,61]. Arayu et al. showed that alpha-fetoprotein-specific CD4+ T-cell responses to three immune-dominant epitopes in HCC patients were significantly expanded during and after embolization (*p* < 0.002). Specifically, the development of alpha-fetoprotein-specific CD4+ T-cells after treatment was significantly associated with the induction of >50% necrosis of tumor and an improved clinical outcome (*p* < 0.007) [57]. Similarly, Mizokushiet al, evaluating T-cell responses in patients with HCC undergoing RFA, observed immune responses to antigens for which no T-cell response was detected before RFA [60]. Interestingly, the number of tumor-specific T-cells after RFA correlated with the prevention of HCC recurrence in patients treated with curative intent [60]. Moreover, RFA ablation not only provides activating signals for T-cell responses against HCC, but also augments the pool of circulating natural killer (NK) lymphocytes and enhances preferential expression of NK cells’ activating receptors and NK cells’ cytotoxicity, and all these effects are seen as soon as one week after treatment [61].

Although very limited data exist in patients with very early or early HCC (BCLC 0 or BCLC A stage) and intermediate HCC (BCLC B stage) treated with ICIs in the adjuvant and neo-adjuvant setting, preliminary data are promising. With regards to the neo-adjuvant setting, a recent pilot randomized, Phase II trial showed that dual treatment with nivolumab plus ipilimumab prior to surgery leads to a complete pathological response rate in 33.3% of cases [62]. An increase in T-cell infiltration and upregulation of cytotoxic and effector memory cell markers in tissue after treatment was also seen, as compared with before treatment [62]. Two other small studies investigated tumor-specific immune responses after combined TACE and RFA treatment, or after each individual treatment, confirming that ablative therapies induce tumor-specific T-cell responses in individual patients upon ablative therapies [59,63].

Combined ICIs plus TACE or RFA are not the only treatments under investigation, as some reports regarding the combination of trans-arterial Y^90^-radioembolization (TARE) and immune checkpoint blockade have been presented at recent oncological meetings with promising results. In particular, Tai et al. reported the results of a Phase II, open-label, single-center, non-randomized trial regarding the effects of a combined therapy with TARE and nivolumab for advanced HCC in an Asian cohort. Their results showed that this combination had a synergistic effect, with an ORR of 30.5% and with good safety and tolerability profiles [64].

Based on these findings, several trials are ongoing to test the efficacy of combined ICIs and locoregional treatments in HCC. This strategy might significantly decrease recurrence rates after treatment with ablative techniques, thus ameliorating long-term prognosis of patients with very early/early HCC. Similarly, ICIs may potentially enhance responses after trans-arterial treatments; this implicates that patients with intermediate stage HCC may be effectively down-staged and might therefore become qualified for curative treatments. Hence, if ongoing studies in this field obtain good results in terms of safety and efficacy, ICIs would not only play a role in the setting of advanced HCC, but would also become a fundamental component of the management of the earlier stages of this tumor.

## 5. Amenability to Atezolizumab Plus Bevacizumab in Real-Life Setting

Given the expected upcoming change in the standard of care for the treatment of patients with advanced HCC, with a preferential use of the combination of atezolizumab plus bevacizumab as first-line treatment, we aimed to explore the actual estimates of the potential applicability in clinical practice of this dual treatment in the Western HCC population. In order to do so, we applied the inclusion and exclusion criteria of the atezolizumab plus bevacizumab IMBrave-150 study to the HCC population recorded in the Italian Liver Cancer (ITA.LI.CA) database. We used this database as it is representative of the real-life setting of HCC patients in Italy: the ITA.LI.CA database, indeed, includes more than 10,000 patients with newly diagnosed or recurrent HCC, with various underlying liver disease etiologies at all stages, who are managed in a large number of Italian centers with different levels of expertise (secondary and tertiary referral centers). Thus, it provides a reliable insight into the characteristics of HCC patients in Western regions and allows for predicting figures of the potential utilization of newly available HCC drugs in real-life clinical practice [39].

**Figure 3:**
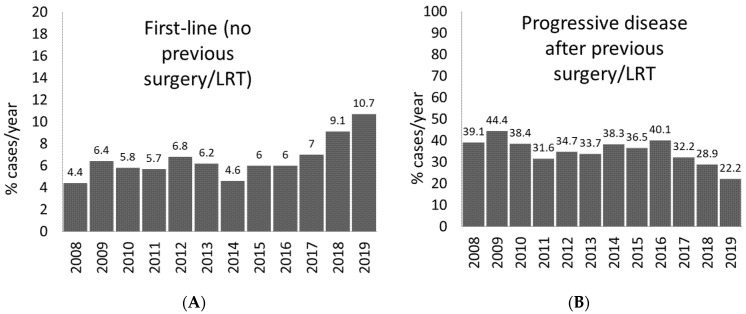
(**A**,**B**) Proportion of patients with new onset HCC or with HCC recurrence after surgery or locoregional treatment, which is amenable to first-line treatment with atezolizumab plus bevacizumab, per year, in the ITA.LI.CA database.

In order to carry this out, within the ITA.LI.CA database, we excluded patients diagnosed before 2008—that is the year of availability of sorafenib in clinical practice in Italy—and we applied the inclusion and exclusion criteria, listed in Table 2, set forth in the Phase III IMbrave-150 trial in patients with advanced HCC. In the studied period (2008–2019), 7529 cases of HCC were reported overall and, among them, a total of 5203 cases had a newly diagnosed HCC, whereas 2326 presented the first recurrence after surgery and/or locoregional treatment; we then calculated the eligibility rate to atezolizumab plus bevacizumab in the overall cohort and, separately, in the two subgroups of naïve patients with HCC or with an HCC recurrence after surgery or locoregional treatment (Figure 3).

As far as the subgroup of naive patients with HCC is concerned, the overall proportion of patients deemed eligible for atezolizumab plus bevacizumab was 7.1%, ranging from 5.3% to 5.4% (2008–2014) up to 10.7% (2019), with a median eligibility rate for the novel therapy in this group of patients of 7.5%, and with an increasing trend observed in the most recent years (Figure 3A). With regard to patients with HCC recurrence after surgery or locoregional treatment, after excluding those not eligible for the treatment with atezolizumab plus bevacizumab as per the study inclusion and exclusion criteria, the overall eligibility rate to this ICI-based therapy was 36.3%, with a median eligibility rate across the whole period of 36.5% (range, 28.9% to 44.4%), with a decreasing trend observed in the most recent years (Figure 3B).

Taking into account all the patients included in the ITA.LI.CA database in the period 2008–2019, irrespective of previous locoregional treatment, approximately 16% of cases were considered eligible for the newly approved dual treatment. This figure is in accordance with estimates from other reports on ICI-based treatments [39].

**Figure 4:**
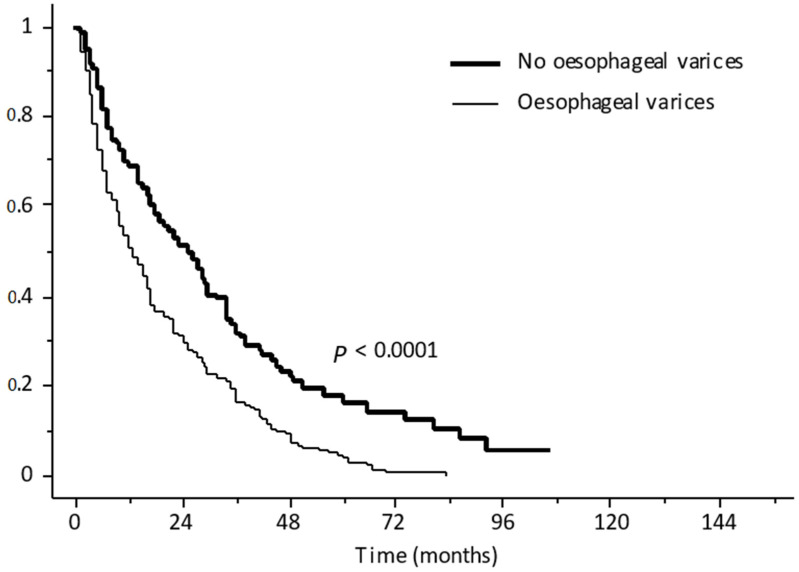
Overall survival of patients with advanced hepatocellular carcinoma, subdivided according to the presence of esophageal varices. Reprinted with permission from ref. [65]. Copyright 2006 American Gastroenterological Association.

Among patients with newly diagnosed HCC, 1.4% of patients were excluded solely due to the presence of untreated, or incompletely treated, esophageal varices at high risk of bleeding, while this figure among patients with recurrence following locoregional treatment or surgery was 4.0%. However, the presence of esophageal varices at high risk of bleeding should not be considered a strict exclusion criterion, as primary prevention of variceal bleeding can and must be performed with either non-selective beta-blockers or endoscopic banding ligation as part of the standard of care of patients with cirrhosis [51,52]. Ligation, which might be preferred due to the possibility of an objective assessment of treatment success, may delay by several weeks the beginning of anti-tumor treatment due to the need to fully evaluate the eradication of varices in a proportion of patients ranging from 1.4% to 4.0%. These considerations need to be taken into account in the therapeutic decision process, as overall approximately 13% of patients with HCC harbor large esophageal varices, a finding keeping with the overall prevalence of at-risk varices in this study population (i.e., 15.0%) before the application of the inclusion/exclusion criteria of the atezolizumab plus bevacizumab study [65]. Moreover, besides representing an issue to be solved before the beginning of treatment, the presence of varices has an inherent meaning that needs to be underscored in these patients, as it pinpoints a subpopulation of patients that—despite having similar inclusion criteria—presents a more advanced liver disease, characterized by clinically significant portal hypertension. This finding is not negligible when patients’ prognosis is assessed, as the presence of esophageal varices is an independent prognostic determinant, also considering the stage of liver disease and HCC stage [51,65,66,67]. Therefore, the prognosis of patients with advanced HCC and esophageal varices will be poorer than that of patients without varices, regardless of the efficacy of the anti-tumoral drug (Figure 4) [65]; as such, screening and treatment (either with band ligation or beta-blockers, selected on a case by case basis) is strongly recommended and must be performed in all patients with HCC, independently from the tumor stage and prior to the initiation of any anti-tumoral treatment.

## 6. Conclusions

Immunotherapy certainly represents a new, exciting frontier in the treatment of advanced, unresectable HCC, and might play a role as an adjuvant or neo-adjuvant treatment of patients with early-stage HCC as well, giving them the chance to decrease the risk of tumor recurrence. New ICI-based treatment strategies with dual, or even triple, combinations of immune-targeting agents, or combinations of immunotherapy and TKIs or other anti-neoplastic agents, will probably be available in the foreseeable future. Thus, it is currently difficult to predict the future algorithm for the systemic treatment of advanced HCC and to state whether sorafenib and lenvatinib, as single agents, will still be listed among the first-line treatment options for this cancer. However, despite the understandable enthusiasm for immunotherapy, some unmet needs remain and require further, extensive research to be resolved. First, as many as 30–40% of patients with HCC do not respond to ICIs, and biomarkers predicting treatment response are lacking. This is a particular challenging issue as data about histological or serological biomarkers related to the effectiveness of ICIs in HCC have not been clearly identified, and, even if a histological marker was identified, biopsy sampling of HCC is not standard clinical practice for this tumor, which is mostly diagnosed on the basis of its radiological hallmarks; therefore, in the future, the role of liver biopsy in HCC might need to be revisited [68]. Secondly, we have shown that in real-life, also taking into consideration previous treatments, only approximately one-tenth to one-third of patients with HCC are eligible for the recently approved combination of atezolizumab plus bevacizumab. Moreover, the safety and utility of immunotherapy in patients with a greater impairment in liver function, such as Child–Pugh class B patients, still has to be demonstrated, as most trials have explored the safety of these drugs in patients with well-preserved liver function (Child–Pugh class A) and, even though some reports have described an acceptable safety profile of some ICIs in Child–Pugh class B patients, consistent data regarding this topic are lacking, so that no strong recommendation can be made in this regard for the time being. Finally, ICIs are highly expensive drugs and this may represent a serious threat to the worldwide treatment implementation in clinical practice, since a large share of patients with HCC are diagnosed in developing countries, where available economic resources cannot support their use [69].

Taken together, the available evidence clearly shows that ICIs are going to play a pivotal role in the treatment of HCC and will improve the prognosis of patients with advanced HCC and, presumably, of those at earlier stages of the disease as well. We can assume that in the foreseeable future the current treatment algorithms will need revisions based on the most recent evidence. However, considering that in real-life settings a high proportion of patients will probably not be eligible for ICI-based regimens, much effort is still needed in order to optimize treatment strategies for patients with advanced, unresectable HCC.

## Figures and Tables

**Table 1:** Ongoing clinical trials with immune checkpoint inhibitors, alone or in combination with other agents, in HCC.

Trial Name	Phase	Line of Treatment	Design	Patients Enrolled	Endpoints	ClinicalTrial.gov	Company	Status
GO30140	I	First-line	Atezolizumab + Bevacizumab (arm A) Atezolizumab + Bevacizumab (arm F1) Atezolizumab (arm F2)	430	Safety, efficacy, pharmacokinetics	NCT02715531	Hoffmann-La Roche	Active, not recruiting
-	I	No restriction	Ramucirumab + MEDI4736 [HCC] (arm C)	114	DLTs	NCT02572687	Eli Lilly & Co/Astra Zeneca	Active, not recruiting
NUANCE	I	Second-line	Nivolumab + bevacizumab	1	Safety and tolerability	NCT03382886	University of Utah	Terminated
-	I	Neo-adjuvant	Nivolumab + cabozantinib	15	Safety and tolerability	NCT 03299946	Sidney Kimmel Compehensive Cancer Center at John Hopkins	Active, not recruiting
-	Ib	First-line	Regorafenib + pembrolizumab	57	Safety and tolerability	NCT03347292	Bayer	Recruiting
-	Ib	First-line	Pembrolizumab + lenvatinib	104	Safety and tolerability	NCT 03006926	Eisai Co., Ltd.	Active, not recruiting
-	Ib	First-line	Nivolumab + lenvatinib	30	Safety and tolerability	NCT03418922	Eisai Co., Ltd.	Active, not recruiting
-	Ib	Second-line	Sintilimab + IBI305	47	AEs/ORR	NCT04401813	Innovent Biologics (Suzhou) Co., Ltd.	Recruiting
-	I/IIa	First-line	Nivolumab + Pexastimogene devacirepvec		Safety and tolerability	NCT03071094	Transgene	Active, not recruiting
CheckMate 040	I/II	Second-line	Cohort 4: Nivolumab + ipilimumab Cohort 6: Nivolumab + cabozantinib	148	Safety and tolerability	NCT01658878	Bristol-Myers Squibb/Ono Pharmaceutical Co., Ltd.	Active, not recruiting
-	I/II	Second-line	SHR-1210 + apatinib	60	OS	NCT02942329	The Affiliated Hospital of the Chinese Academy of Military Medical Sciences	Unknown
-	Ib/II	First-line	Pembrolizumab + talimogene laherarepvec	244	ORR/DLTs	NCT02509507	Amgen	Recruiting
-	II	First-line and Second-line	Durvalumab + tremelimmumab [regimen 1] (arm A) Durvalumab (arm B) Tremelimumab (arm C) Durvalumab + tremelimumab [regimen 2] (arm D) Durvalumab + bevacizumab (arm E)	545	Safety and tolerability	NCT02519348	MedImmune, LLC	Active, not recruiting
RESCUE	II	Second-line	SHR-1210 + apatinib	190	ORR	NCT03463876	Jiangsu HengRui Medicine Co., Ltd.	Active, not recruiting
-	II	First-line/Second-line	SHR1210 + apatinib (arm A) SHR1210 + FOLFOX4 or GEMOX regimen (arm B)	152	Safety and tolerability	NCT03092895	Jiangsu HengRui Medicine Co., Ltd.	Unknown
IMMUNIB	II	First-line	Nivolumab + lenvatinib	50	ORR/safety and tolerability	NCT03841201	Institut fur Klinische Krebsforschung IKF GmbH	Recruiting
-	II	First-line/Second-line	Nivolumab + Ipilimumab vs. nivolumab		Safety and tolerability	NCT03222076	MD Anderson Cancer Center	Active, not recruiting
-	II/III	First-line	Sintilimab + IBI305	566	OS/PFS	NCT03794440	Innovent Biologics (Suzhou) Co., Ltd.	Recruiting
IMbrave150	III	First-line	Atezolizumab + bevacizumab (arm A) Sorafenib (arm B)	480	OS/PFS	NCT03434379	Hoffmann-La Roche	Active, not recruiting
COSMIC-312	III	First-line	Cabozantinib + atezolizumab (arm A) Sorafenib (arm B) Cabozantinib (arm C)	740	PFS/OS	NCT03755791	Exelixis	Recruiting
LEAP-002	III	First-line	Pembrolizumab + Lenvatinib vs. placebo + lenvatinib	750	PFS/OS	NCT03713593	Merck Sharp & Dohme Corp.	Active, not recruiting
-	III	First-line	SHR-1210 + FOLFOX4 vs. sorafenib or FOLFOX4	448	OS	NCT03605706	Jiangsu HengRui Medicine Co., Ltd.	Recruiting
HIMALAYA	III	First-line	Durvalumab (arm A) Durvalumab + tremelimumab [regimen 1] (arm B) Durvalumab + tremelimumab [regimen 2] (arm C) Sorafenib (arm D)	1310	OS	NCT03298451	AstraZeneca	Active, not recruiting
-	III	First-line	CS1003 + lenvatinib vs. placebo + lenvatinib	525	PFS/OS	NCT04194775	CStone Pharmaceuticals	Recruiting

HCC, hepatocellular carcinoma; DLTs, dose-limiting toxicities; AEs, adverse events; OS, overall survival; PFS progression-free survival; ORR, overall response rate.

**Table 2:** Criteria of eligibility for the management of unresectable HCC with atezolizumab plus bevacizumab as a first-line therapy.

IMBrave-150 Inclusion Criteria
Age ≥ 18 years
Locally advanced or metastatic and/or unresectable HCC
No prior systemic therapy for HCC
Disease that is not amenable to curative surgical and/or locoregional therapies, or progressive disease after surgical and/or locoregional therapies
At least one measurable (per RECIST 1.1) untreated lesion
Patients who received prior local therapy (e.g., radiofrequency ablation, percutaneous ethanol or acetic acid injection, cryoablation, high-intensity focused ultrasound, transarterial chemoembolization, transarterial embolization, etc.) are eligible provided the target lesion(s) have not been previously treated with local therapy or the target lesion(s) within the field of local therapy have subsequently progressed in accordance with RECIST version 1.1
ECOG PS 0-1
Child–Pugh class A
ANC ≥ 1.5 × 10^9^/L (1500/mcL) without granulocyte colony-stimulating factor support
Lymphocyte count ≥ 0.5 × 109/L (500/µL)
Platelet count ≥ 75 × 109/L (75,000/µL) without transfusion
Hemoglobin ≥ 90 g/L (9 g/dL)
AST, ALT, and alkaline phosphatase (ALP) ≤ 5 × upper limit of normal (ULN)
Serum bilirubin ≤ 3 × ULN
Serum creatinine ≤ 1.5 × ULN or creatinine clearance ≥ 50 mL/min (Cockcroft–Gault formula)
Serum albumin ≥ 28 g/L
For patients not receiving therapeutic anticoagulation: INR or aPTT ≤ −2 × ULN
Urine dipstick for proteinuria < 2+
Negative HIV test at screening
In case of active HBV, HBV DNA < 500 IU/mL and anti-HBV treatment for a minimum of 14 days prior to study entry
No history of leptomeningeal disease
No active or history of autoimmune disease or immune deficiency
No history of idiopathic pulmonary fibrosis, organizing pneumonia, drug-induced pneumonitis, or idiopathic pneumonitis, or evidence of active pneumonitis
No active tuberculosis
No significant cardiovascular disease (≥NYHA Class II)
No major surgical procedure, other than for diagnosis, within 4 weeks
No history of malignancy other than HCC within 5 years prior to screening
No severe infection within 4 weeks prior to initiation of study treatment
No treatment with therapeutic oral or IV antibiotics within 2 weeks prior to initiation of study treatment
No prior allogeneic stem cell or solid organ transplantation
No known fibrolamellar HCC, sarcomatoid HCC, or mixed cholangiocarcinoma and HCC
No untreated or incompletely treated varices with bleeding or high risk for bleeding
No moderate or severe ascites
No history of hepatic encephalopathy
No co-infection of HBV and HCV
No symptomatic, untreated, or actively progressing central nervous system (CNS) metastases
No uncontrolled pleural effusion, pericardial effusion, or ascites requiring recurrent drainage procedures
No uncontrolled or symptomatic hypercalcemia
No treatment with systemic immunosuppressive medication
No inadequately controlled arterial hypertension
No significant vascular disease
No history of intra-abdominal inflammatory process

## Data Availability

Data available on request due to restrictions. The data presented in this study are available on request from the corresponding author. The data are not publicly available due to the rules of the ITA.LI.CA consortium.
